# Liposome-Coated Iron Fumarate Metal-Organic Framework Nanoparticles for Combination Therapy

**DOI:** 10.3390/nano7110351

**Published:** 2017-10-26

**Authors:** Bernhard Illes, Stefan Wuttke, Hanna Engelke

**Affiliations:** 1 Department of Chemistry and Center for NanoScience (CeNS), LMU Munich, Butenandtstraße 11 (E), 81377 Munich, Germany; bernhard.illes@cup.uni-muenchen.de; 2 School of Chemistry, Joseph Banks Laboratories, University of Lincoln, LN67TS Lincoln, UK

**Keywords:** metal-organic framework, nanoparticles, drug delivery, combination therapy

## Abstract

One of the main problems for effective treatment of cancer is resistances, which often require combination therapy—for effective treatment. While there are already some potential drug carriers—e.g., liposomes, available for treatment—the effective loading and retention of the desired drug ratio can be challenging. To address this challenge, we propose a new type of drug carrier: liposome-coated metal-organic framework (MOF) nanoparticles. They combine the advantages of liposomes with an easy and efficient loading process. In this work, we present the successful synthesis of liposome-coated MOF nanoparticles via the fusion method. The resulting particles, once loaded, show no premature leakage and an efficient release. Their successful loading with both single and multiple drugs at the same time makes them an interesting candidate for use in combination therapy.

## 1. Introduction

Metal-organic frameworks (MOFs) are a uniquely valuable class of materials, in that their structure and composition can be controlled at the atomic level. As the name suggests, MOFs are made of metal ions, or clusters (inorganic), and organic molecular building units, assembling together into crystalline solids [1,2,3,4]. This construction from building units and the nearly endless pool of different organic and inorganic units to choose from give MOFs their incredible chemical flexibility, and give us control on the atomic level. Each possible building unit contributes its own properties and features, which allows us to make MOFs that have very specific degrees of porosity (pore and window size), surface area, functionality and biocompatibility [5,6]. All these attributes make them highly interesting to develop nanostructured smart drug delivery systems capable of bypassing extra- and intracellular barriers [7,8,9,10,11,12,13,14,15,16,17,18].

In the last 5 years, a number of pioneering studies have been reported that highlight the suitability of MOF nanocarriers as a new type of platform for drug delivery [9,19,20,21,22,23,24,25,26,27]. So far, these reports have mainly focused on the delivery of single active agents (e.g., one drug), whereas their application to deliver “cocktails” of drugs is still largely unexplored [7,28].

Current chemotherapy faces the challenge that tumours quickly become resistant to a drug during treatment. One possible solution to this problem is the administration of multiple drugs at once to fight resistant cancer strains and prevent formation of new resistances [29,30,31,32]. This combination therapy has proven to be more effective than single-drug therapies, but faces the challenge that each drug has different physicochemical properties, which leads to heterogeneous pharmacokinetics and tissue distribution.

In this respect, the use of nanocarriers opens up the possibility of co-encapsulating multiple drugs, and thus synchronising their delivery to the cancer cells [29,30,31]. MOF nanoparticle platforms are especially interesting due to their hybrid nature, relying on the synergistic combination of inorganic and organic chemistry [7]. This allows the creation of chemically diverse internal pore systems able to incorporate drugs with different physicochemical properties. First pioneering studies reporting on such MOF platforms for the delivery of several drugs are very encouraging [32,33,34,35]. One of them even reports on an enhanced efficiency in tumour reduction due to dual drug delivery with MOF nanoparticles [35]. While this study shows great promise, the employed nanoparticles were not fully encapsulated. Such an encapsulation would be desirable, though, to prevent the observed drug leakage and to enhance the stability [36,37].

In this paper, we demonstrate that MOF nanoparticles can be simultaneously loaded with multiple drugs. Furthermore, the drug carriers can then be coated with a lipid shell acting as a temporary seal for the encapsulated drugs, and allowing control of interactions with intracellular fluids. The successful synthesis of liposome-coated MOF nanoparticles is based on a simple fusion method. The resulting particles, once loaded, show no premature leakage. As opposed to a previous study [24], the MOF nanoparticles presented here also show an efficient intracellular release. In our study we focus on iron-based MOF nanoparticles, namely MIL-88A, which are composed of iron(III) and fumaric acid, both naturally occurring in the body [38]. These particles were loaded with Suberoyl bis-hydroxamic acid (SBHA) alone, or the two drugs irinotecan and floxuridine together. The two latter drugs were chosen because past studies have shown an improved efficacy in preclinical tumour models [39], making them interesting candidates for the use in combination therapy. Liposomes loaded with both drugs in a 1:1 ratio are currently in an ongoing clinical trial under the name CPX-1 [40].

## 2. Results and Discussion

### 2.1. MOF Nanoparticle Synthesis and Their Lipid Coating

MIL-88A nanoparticles were synthesized using a microwave approach before being loaded and then coated with DOPC (1,2-dioleoyl-sn-glycero-3-phosphocholine) derived liposomes using the fusion method after loading [41] (see Supplementary Materials for characterisation). The liposome-coated MIL-88A nanoparticles, further referred to as Lip-MIL-88A, was used to carry several drugs and investigated towards its loading capacity, release behaviour and effectiveness at loading multiple drugs at once and thus a possible application in combination therapy. 

### 2.2. Loading Capacity

**Figure 1:**
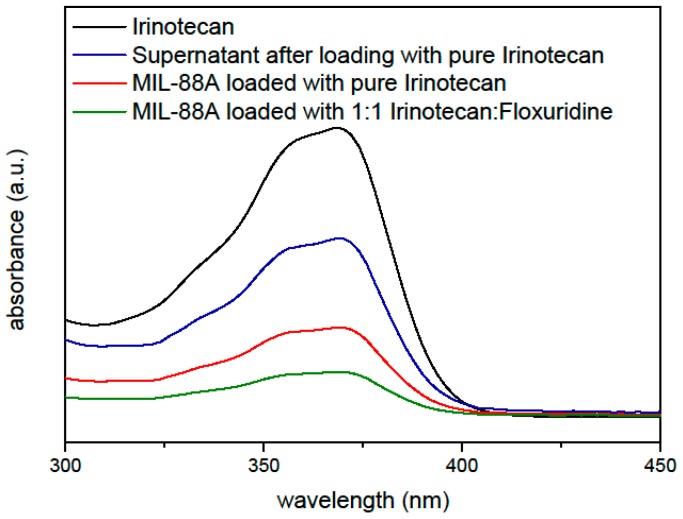
UV/Vis measurements to determine the loading capacity of Lip-MIL-88A nanoparticles for Irinotecan.

The first step was to investigate the drug loading capacity of MIL-88A. For this purpose, MIL-88A nanoparticles were loaded with irinotecan and floxuridine in different ratios through soaking in 1 mM aqueous solutions of the biotherapeutics, before coating them with liposomes. These particles were then investigated via UV/Vis spectroscopy (Figure 1) to determine the loading capacity. The absorption maximum of irinotecan at 360 nm was used as the basis for the calculations. Particles loaded with different ratios of irinotecan were examined and compared to the UV/Vis spectrum of a pure irinotecan solution to calculate the amount of drug loading. For this 1 mg of MIL-88A nanoparticles were suspended in 1 mL of a 1 mM solution of irinotecan. The amount of irinotecan in the MIL-88A nanoparticles was quantified by UV/Vis measurements, yielding a loading capacity of 21 wt % relative to the nanoparticle weight (205 µg) for loading only irinotecan, and 10.3 wt % (102 µg) of irinotecan when a 0.5 mM irinotecan and 0.5 mM floxuridine solution (further referred to as 1:1) is used for the loading. Similarly, the loading capacity of floxuridine was determined to be 3.61 wt % for a 1 mM solution of floxuridine only and in the mixtures with irinotecan it was reduced by the respective factor of dilution (measurements see Supplementary Materials). Thus, the nanoparticles can successfully be loaded with both drugs at the same time yielding a ratio of the mixture as provided in the loading solution weighted by the loading capacity of the pure drugs.

### 2.3. Fluorescence Release Experiments

**Figure 2:**
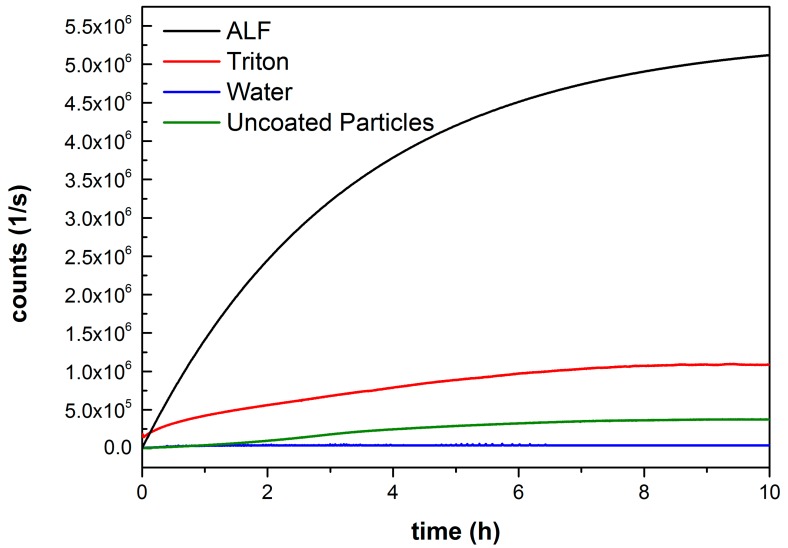
Fluorescence release measurement of Lip-MIL-88A in various solvents. Water (**blue**) and water with addition of Triton X-100 (**red**), as an additional control, as well as ALF (**black**), to simulate the environment of the lysosome. As proof that the liposomes prevent the leakage of the cargo, uncoated particles (**green**) were also measured.

To further investigate the loading and especially the release behaviour of the Lip-MIL-88A particles, as well as to test for possible leakage of the cargo from the particles, fluorescence release experiments were performed (Figure 2). The particles were loaded with the membrane impermeable dye calcein as a fluorescence marker. An aqueous solution of the calcein-loaded Lip-MIL-88A was used as a control and a test for possible leakage. To guarantee a release, a second sample was treated with triton X-100 dissolving the liposome coating. In addition, artificial lysosomal fluid (ALF) [42] was used to simulate the environment the particles encounter in the lysosome after entering the cell. Finally, the uncoated particles were also measured in water. The coated particles in pure water show no release even after several hours, while the uncoated particles exhibit a fluorescence increase and thus leakage, proving the effectiveness of the liposome coating in preventing side effects caused by leakage. Both the triton X-100 and ALF treated samples show a clear release of the calcein from the particles. The large difference in the strength of the release is caused by the complete dissolution of the particle through the ALF in comparison to only opening the membrane in the case of triton X-100. This dissolution of the particles in ALF leads to a full release of the cargo inside the cell [10]. The difference between the uncoated particles and those treated with triton X-100 can be explained by the lipid coating. For the uncoated particles, a steady release of calcein is observable, as the calcein is released without hindrance. That the triton X-100-treated particles show a strong release in the beginning is most likely due to the calcein detaching from the MOF, but being contained by the lipid membrane, as soon as the membrane is punctured, this already-freed calcein is released at once, leading to the strong initial increase in fluorescence. The difference in the amount of released dye in saturation might be either due to more efficient encapsulation facilitated by the lipid bilayer, or due to triton possibly interfering with the interaction between particle and calcein leading to an enhanced release. The release behaviour of the investigated drugs might differ from that of calcein due to its carboxylate groups, which allow it to attach to the surface of the MOF, and might lead to a slower release. The general behaviour of the particles in the different media should, however, stay unaffected.

### 2.4. Cell Release Experiments

**Figure 3:**
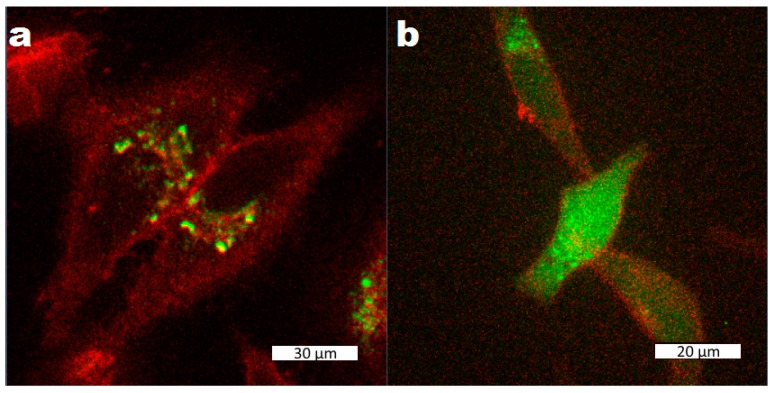
Liposome-coated MIL-88A nanoparticles loaded with calcein after two (**a**) and three (**b**) days of incubation with HeLa cells. The cells have been marked with CellMask Orange. The scale bars correspond to 30 µm (**a**) and 20 µm (**b**).

To investigate the cell uptake of the nanoparticles and their drug release behaviour inside cells, especially if the strong release observed after exposure to ALF also takes place in the actual cell environment, Lip-MIL-88A particles were again loaded with calcein as a model drug and incubated in HeLa cells. As can be seen in Figure 3, after two days, the particles were taken up by the cells, but no release of the calcein could be observed yet. After three days of incubation, release of calcein from the particles without any outside stimulus could be observed. Thus Lip-Mil-88A avoids any dangers caused by potentially harmful release triggers, reducing the potential side effects. The observed release can be explained by the results from the fluorescence release experiments: The particles are taken up via endocytosis and when they are not yet in the lysosomal environment, they show no release. Once they are in the lysosomal environment, the particles dissolve, leading to a release of their cargo [10].

### 2.5. Single Drug Cytotoxicity (MTT) Assays

**Figure 4:**
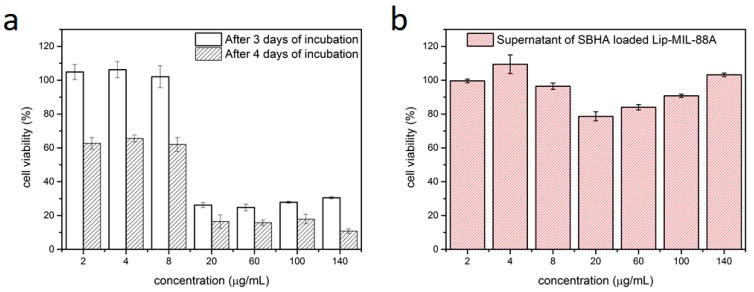
(**a**) MTT assay of HeLa cells incubated with Lip-MIL-88A nanoparticles loaded with SBHA after three and four days of incubation. (**b**) MTT assay of HeLa cells incubated with the supernatant of the Lip-MIL-88A suspension after four days. The error bars represent the standard deviation.

The liposome-coated MIL-88A nanoparticles were loaded with SBHA, a histone deacetylase inhibitor, which leads to cell death, as a model drug and to further test its drug-carrying capabilities. The particles were then incubated in HeLa cells for 3 to 4 days and MTT-assays were performed (Figure 4a). The SBHA-loaded Lip-MIL-88A proved effective against the HeLa cells, showing cell viabilities of only 15% after 4 days of incubation. The particles proved to be able to safely deliver their cargo to the target cells showing an IC_50_ of about 15 µg/mL of SBHA loaded Lip-MIL-88A. To investigate if the cell deaths could be caused by leakage of SBHA from the drug carrier, the supernatant was collected and also investigated via MTT-assay (Figure 4b). These measurements show that no leakage occurred, as the cells are still viable even at high concentrations of the supernatant. The loading capacity of this system for SBHA was determined to be 2.90 wt % with respect to the particles (see Supplementary Materials). This further corroborates the general suitability of Lip-MIL-88A as a drug delivery vehicle.

### 2.6. Multi Drug MTT-Assays

Next, the particles where loaded with both irinotecan and floxuridine in different ratios (Table 1) to determine both the effectiveness of each single drug loaded on the particles and the effectiveness of the mixtures. For this purpose, particles loaded with pure irinotecan and floxuridine and different ratios of both (1:3, 1:1 and 3:1, with the ratios referring to the molar ratios of the drugs in the loading solution) were incubated with cells. The MTT assays show that both irinotecan and floxuridine could be successfully loaded into the Lip-MIL-88A, leading to a significantly reduced cell viability of 34.6% with an IC_50_ of 4 µg/mL for the irinotecan-loaded version. Floxuridine had a significantly lower efficiency, which did not even reach a 50% reduction of cell viability within the measured amounts of particle administration. The mixtures of them were also tested (Table 1), with the 3:1 ratio showing the best results achieving a low cell viability similar to irinotecan alone. All tested ratios aside from pure floxuridine reduce the cell viability to 30–40% at the highest tested concentration of 140 µg/mL, while pure floxuridine only manages to reach a reduction to 60% at this particle concentration. This is in accordance to the 1:3 irinotecan:floxuridine ratio being the least effective of the investigated mixtures.

**Figure 5:**
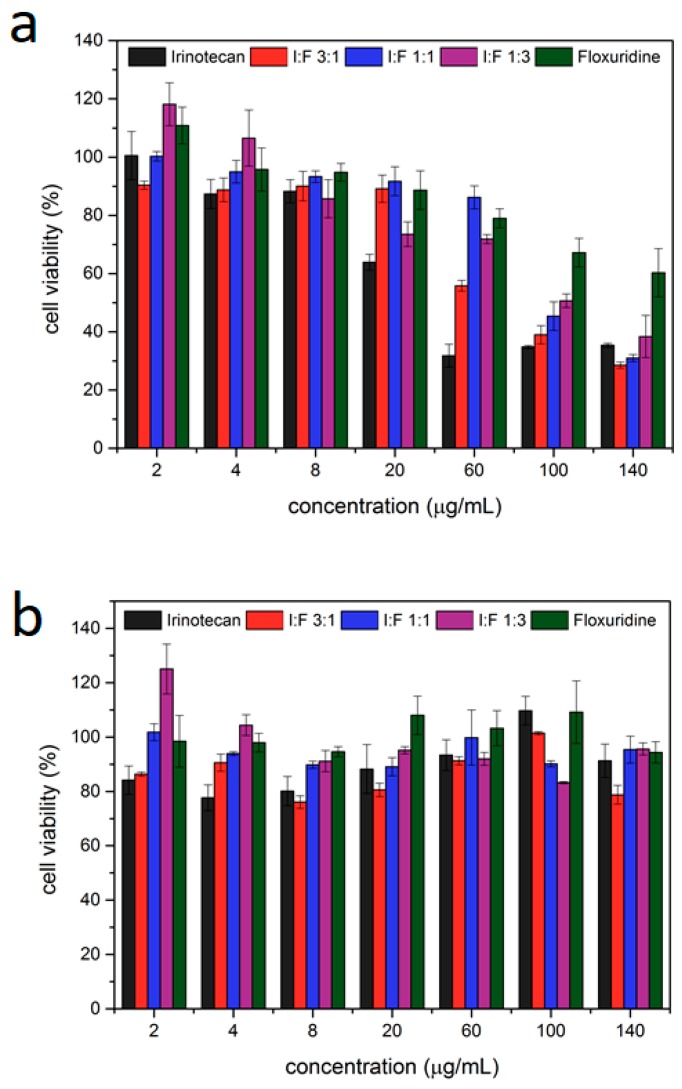
(**a**) MTT-assay of Lip-MIL-88A particles loaded with different ratios of irinotecan/floxuridine incubated in HeLa cells for three days. The error bars represent the standard deviation. (**b**) MTT-assay of the supernatant of Lip-MIL-88A particles loaded with different ratios of irinotecan/floxuridine after incubation in HeLa cells for three days. The error bars represent standard deviations.

To rule out leakage of the drugs as the responsible factor for the observed reduction in cell viability, the particles were stored in PBS for several days before the supernatant was collected and tested for its toxicity (Figure 5b) showing no significant decrease in cell viability. With this, we can exclude drug leakage from the particles as the reason for the observed cell death. Thus, the liposome-coated MOF nanoparticles can serve as a drug delivery system for dual therapy preventing leakage and taking advantage of the delivery of two different drugs. Although the combinations of drugs yield lower efficiencies than irinotecan alone due to the lower efficiency of floxuridine, the option of dual drug delivery with these nanoparticles shows great promise in the fight against resistances via combinatorial drug application.

## 3. Conclusions

In conclusion, the Lip-MIL-88A platform is a promising tool for use as a platform for combination therapy. It could readily take up a significant amount of biologically active agents (20 wt %) and showed a promising release of its cargo. Due to the liposome coating, no leakage of the cargo could be observed. In cell experiments, Lip-MIL-88A nanoparticles were readily taken up by cells and showed a significant intracellular release after three to four days of incubation. Different drugs (SBHA, irinotecan and floxuridine) were successfully loaded into the particles, and the drug delivery system enables intracellular release profiles. The successful and uncomplicated loading, as well as the effective intracellular release of drugs compared to normal liposome loading procedures, is a promising start for future applications in combination therapy and could as such contribute to improve cancer chemotherapy.

## 4. Materials and Methods

All chemicals were purchased from Sigma Aldritch (St. Louis, MO, USA) unless noted otherwise.

### 4.1. UV/Vis Measurements

The UV/Vis measurements were performed on a Lambda 1050 UV/Vis/NIR spectrometer form Perkin Elmer (Waltham, MA, USA). The software used to record the measured spectra was Perkin Elmer UVWinLab (Waltham, MA, USA). For the measurements, the loaded coated particles were dissolved in ALF to facilitate the release of all their cargo. 1 mL of this solution was then diluted with 2 mL H_2_O yielding a total of 3 mL and measured in a quartz cuvette. For the pure drugs, 1 mL of the aqueous solution was diluted with 1 mL H_2_O and 1 mL ALF.

### 4.2. Fluorescence Microscopy

The fluorescence microscope images were recorded with a Zeiss Observer SD (Jena, Germany) spinning disk confocal microscope using a Yokogawa (Musashino, Japan) CSU-X1 spinning disc unit and an oil objective with 63× magnification and BP 525/50 and LP 690/50 filters. The setup was heated to 37 °C and a CO_2_ source was provided to keep the atmosphere at 5% CO_2_. For both excitation of the calcein and the cell marker a laser with a wavelength λ = 488 nm was used. The images were processed with the Zen software by Zeiss to optimize contrast.

### 4.3. Fluorescence Spectroscopy

The fluorescence spectroscopy experiments were recorded with a MD-5020 setup from PTI Photon Technology International (Birmingham, UK). The software Felix32 was used for recording and evaluating the measured data. For the experiments hollow caps were filled with 50 µL of a 1 mg/mL particle stock solution. Depending on the experiment, 100 µL of water or ALF or 90 µL water and 10 µL 20% triton X-100 solution were added, before the caps were sealed with a dialysis membrane and placed into cuvettes filled with water together with a stirring rod. The measurement temperature was 37 °C, with an excitation wavelength of 495 nm and an emission wavelength of 512 nm.

### 4.4. Cell Culture

All cell experiments were prepared in a Hera-Safe cell culture unit from Heraeus (Hanau, Germany). The cells were incubated at 37 °C/5% CO_2_ in Hera Cell incubators also from Heraeus. Cells were grown in Dulbecco’s modified eagle medium (DMEM) with 10% fetal bovine serum (FBS) and 1% PenStrep. These chemicals were purchased from Thermofisher Scientific (Waltham, MA, USA).

### 4.5. MTT Assays

The MTT-assays were performed with a Spectra Fluor Plus from Tecan (Männedorf, Switzerland) and were then evaluated with Excel 2010 and Origin. 5000 cells were seeded per well and incubated together with 100 µL DMEM (10%FBS, 1% PenStrep) for 1 day before the particles were added. Each concentration was tested on three different days and on each day in triplicate. After the allotted incubation time the plates were washed with Hank’s Balanced Salt solution (HBSS) to remove dead cells, before the MTT reagent diluted in DMEM (0.5 mg/mL) was added. After two hours of incubation the MTT reagent solution was removed from the cells, and the cells were frozen at −80 °C for 0.5 h. Before the measurement 100 µL dimethyl sulfoxide (DMSO) were added to each well.

### 4.6. Synthesis of the Uncoated and Coated MIL-88A Nanoparticles

#### 4.6.1. Synthesis of MIL-88A NPs

MIL-88A nanoparticles were synthesized in a microwave assisted approach based on the results of Chalati et al. [38]. In this synthesis route, an aqueous solution of FeCl_3_·6H_2_O (1.084 g, 4.01 mmol) and fumaric acid (485 mg, 4.18 mmol) are given to water (20 mL, Milli-Q). The reaction mixture was stirred until the metal salt was completely dissolved. The reaction mixture was then given into a Teflon tube (80 mL) and placed into a microwave oven (Synthos 3000, Anton-Paar, (Graz, Austria)) along with 3 additional vessels. Two of these vessels are filled with water (20 mL, Milli-Q), the third vessel is filled with an aqueous FeCl_3_ (20 mL, 1.084 g, 4.01 mmol) and is used to monitor the reaction progress. The vessels were heated under stirring with the sequence shown in Table 2.

To remove residual reactants, the sample was subsequently washed via centrifugation (7840 rpm, 20 min) and redispersion of the pellet in ethanol (20 mL). This washing cycle was repeated 3 additional times. To also remove bulk material formed during the reaction, the dispersion was then centrifuged 3 times (3 min, 3000 rpm) and the pellet fraction of the product discarded. 

#### 4.6.2. Preparation of the Liposome Coating Solution

A 1 mg/mL PBS solution of DOPC was prepared and extruded through an extruder with a 100 nm pore sized membrane 11 times for cleaning.

#### 4.6.3. Preparation of the Loaded and Coated Particles

1 mg of MIL-88A NPs were suspended in 1 mL of a 1 mM solution of calcein, suberohydroxamic acid (SBHA), irinotecan or floxuridine and incubated overnight for loading. Next, they were centrifuged for 5 min at 14,000 rpm, to discard the supernatant and the pellet was dissolved in 0.2 mL of the liposome coating solution and 0.2 mL water and incubated for 2 h. The particles were then centrifuged (5 min at 14,000 rpm) and redispersed in 1 mL PBS after washing several times.

For the preparation of the particles loaded with both irinotecan and floxuridine the MIL-88A particles were immersed in mixtures of 1 mM irinotecan and floxuridine solutions in the desired ratios, before following the same coating and washing procedure as outlined above.

## Figures and Tables

**Table 1:** Comparison of the different MIL88A loadings and their IC_50_ values.

	Irinotecan	3:1 Iri Floxu	1:1	1:3 Iri Floxu	Floxuridine
Cell viability at 140 µg/mL (%)	34.6	28.6	30.6	37.9	60.5
I_c_ 50 (µg/mL)	40	80	80	80	120

**Table 2:** Microwave heating program for the MIL-88(A) NPs synthesis.

Heating	Dwelling	Cooling
30 s	5 min	45 min
To 80 °C	80 °C	To room temperature

## Supplementary Materials

The following are available online at http://www.mdpi.com/2079-4991/7/11/351/s1.
