# Supplementary material for: Liposome-Coated Iron Fumarate Metal-Organic Framework Nanoparticles for Combination Therapy
Source: Nanomaterials (Basel). 2017 Oct 26;7(11):351. doi: 10.3390/nano7110351 (PMC5707568; doi:10.3390/nano7110351)
Supplement: Supplementary file 1 [file nanomaterials-07-00351-s001.pdf]

Supporting information

Liposome-Coated Iron Fumarate Metal-Organic Framework Nanoparticles for Combination Therapy

Bernhard Illes [1], Stefan Wuttke [1,2,*] and Hanna Engelke [1,*]

1 Department of Chemistry and Center for NanoScience (CeNS), LMU Munich, Butenandtstraße 11 (E), 81377 Munich, Germany; Bernhard.illes@cup.uni-muenchen.de
2 School of Chemistry, Joseph Banks Laboratories, University of Lincoln, LN67TS Lincoln, UK
* Correspondence: stefan.wuttke@cup.uni-muenchen.de (S.W.); hanna.engelke@cup.lmu.de (H.E.); Tel.: +49-89-218077626

Methods

X-Ray Diffraction

X-ray diffraction experiments were performed on the initial MIL-88A NPs. The samples were measured on a STOE Transmissions-Diffraktometer System STADI P operating in transmission mode. The setup is using $CuK_{\alpha 1}$-radiation with a wavelength λ = 015418 nm. The resulting diffraction pattern was evaluated using the software package WinXPOW RawDat v3.0.2.5 and WinXPOW PowDat_n v3.0.2.7.

Scanning Electron Microscopy (SEM)

All SEM micrographs were recorded with a Helios NanoLab G3UC (FEI) operating at 5 kV. During sample preparation an ethanolic NP dispersion was dried on a carbon film placed on an aluminum sample holder. The sample was stored overnight to evaporate the solvent followed by carbon sputtering prior to the measurement. For evaluation of the SEM micrographs the software ImageJ v1.49 was used.

Transmission Electron Microscopy (TEM)

The TEM micrographs of the sample particles were taken on a Titan Themis (Fei) that was operated at an acceleration voltage of 300 kV. For sample preparation, an ethanolic NP solution was dried overnight on a carbon-coated copper grid.

Thermogravimetric Analysis (TGA)

A dried sample of MIL-88A NPs (3.125 mg) was heated using a TASC 414/4 (Netzsch). The sample was heated under synthetic air at 10 °C/min up to 900 °C. The resulting data was evaluated using the software Proteus v4.3.

Nitrogen sorption

Nitrogen sorption experiments were conducted with an Autosorb-1 (Quantachrome). Prior to the measurement the sample (27.9 mg) was outgassed under high vacuum at 120 °C for 38 h. The resulting data was evaluated with the software ASiQwin v3.0. The linearized form of the BET equation was used to calculate BET surface areas. For the calculation of the pore size distribution a QSDFT equilibrium based model was used assuming slit and cylindrical pores.

Dynamic Light Scattering (DLS)

DLS was performed on a Zetasizer Nano Series (Nano-ZS, Malvern) equipped with a laser with the wavelength λ = 633 nm. For sample preparation the freshly prepared nanoparticles (NPs) were dispersed in ethanol or phosphate-buffered saline (PBS) in the case of the capped NPs.

XRD

The XRD measurement of the MIL-88A nanoparticles shows a successful synthesis of the particles, showing the characteristic reflexes at 10-11.

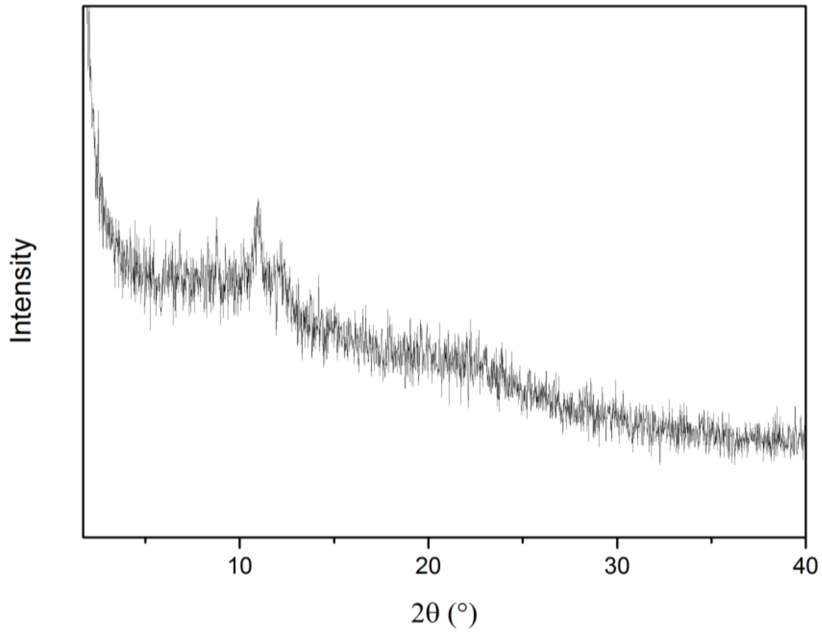

Figure S1: XRD measurement of MIL-88A nanoparticles as synthesized

Scanning Electron microscopy (SEM)

SEM measurements show that the MIL-88A nanoparticles possess a round morphology and are fairly homogenous (Figure S2) featuring a particle size of about 50 nm.

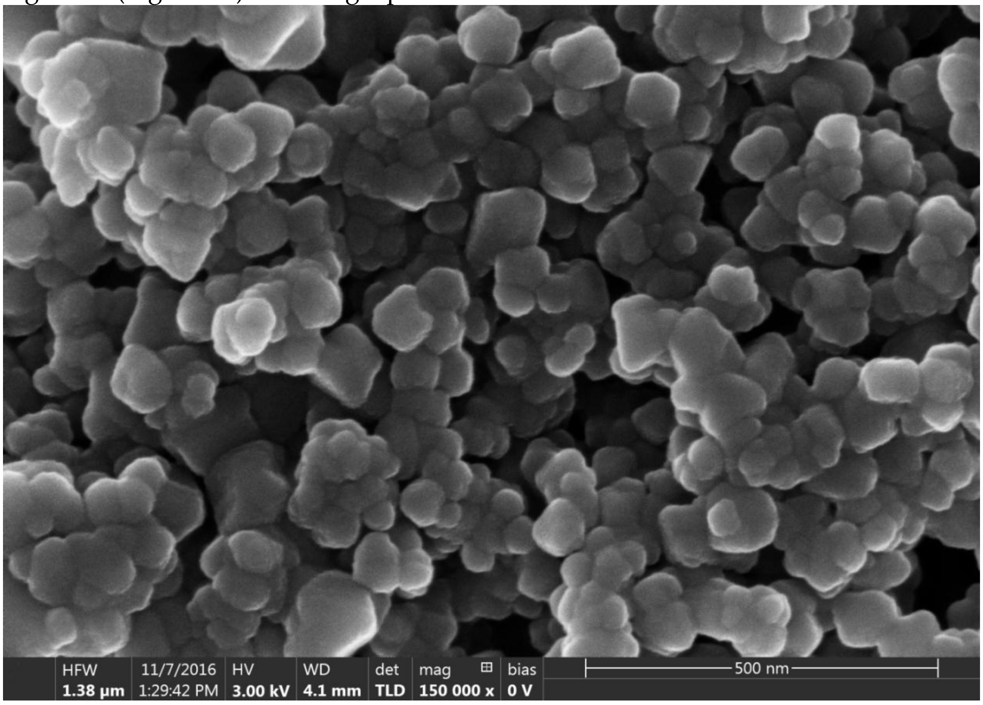

Figure S2: SEM image of MIL-88A NPs.

Transmission Electron Microscopy (TEM)

Figure S3 depicts a TEM micrograph of the MIL-88A NP sample. The particles feature a round morphology and are connected via thin necks. The MIL-88A sample appears to be fairly homogenous.

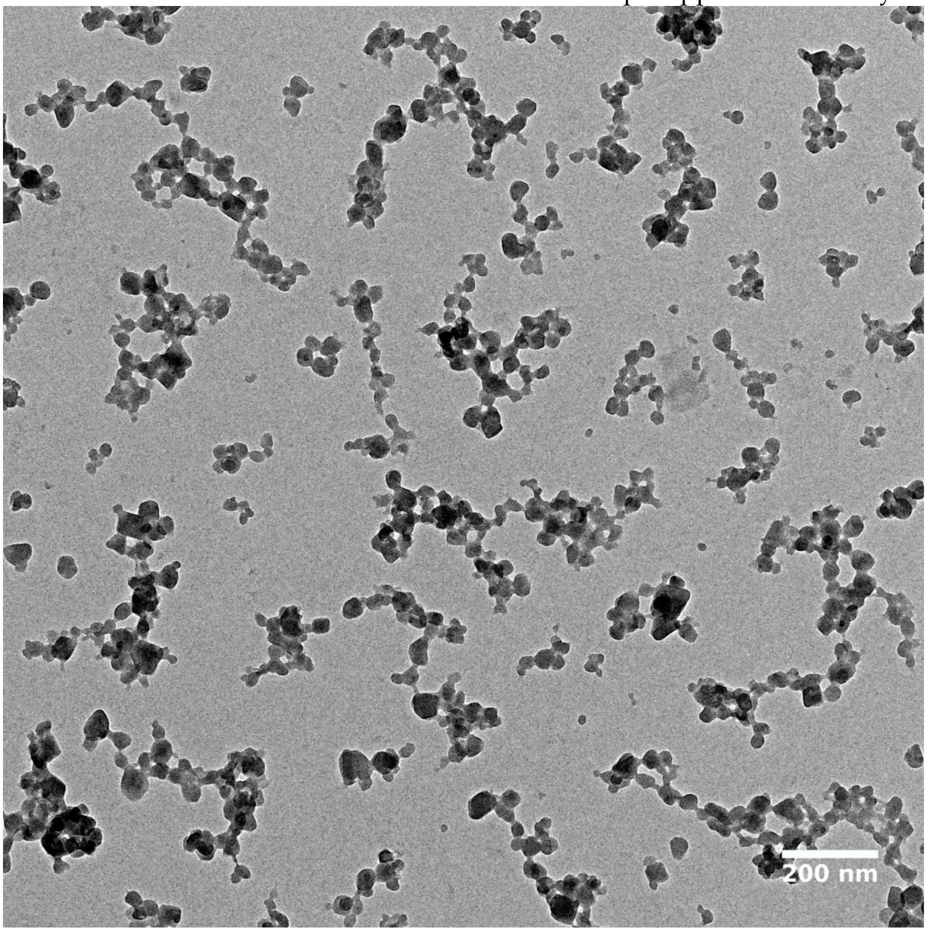

Figure S3: Transmission electron micrograph of MIL-88A NPs.

Thermogravimetric Analysis and Nitrogen Sorption

Thermogravimetric analysis of the synthesized MIL-88A was performed. Up to 204 °C the residual solvent in the sample is desorbed resulting in a mass loss of 6%. Subsequently, in a range between 204–433 °C the framework decomposes. The residual mass left of the sample remains at 42%. This data is in agreement with literature data for MIL-88A NPs and shows the successful synthesis of the MOF.[1] The Nitrogen Sorption gave a BET surface area of 218 m^2/g and a pore size of 11.44 Å, which agrees with literature. [2] Further information can also be found in [3]

Calculating the quenching adjustment factor for the Fluorescence Release Experiments

To account for quenching 10 mL of a 1 mM solution of calcein were diluted with 990 mL PBS. Next, 10 mL of this new solution were added to 4 mL water or ALF in cuvettes. In addition 10 mL triton X-100 was added to one sample. Then the fluorescence of these solutions was measured. The obtained values were used to calculate the offset for the individual fluorescence measurements. The normalization factor determined by comparing the measurements of water and triton X-100 to those of ALF is 2.17 and was implemented in the fluorescence experiments.

UV/Vis of Floxuridine

To determine the loading capacity of floxuridine and the floxuridine content when loading both irinotecan and floxuridine together, UV/Vis measurements were taken. As the irinotecan signal overlays the floxuridine signal at high concentrations the loading for the I:F 3:1 mixture could not be measured.

The floxuridine stock solution contained 1 mM (246 µg) floxuridine. Using this as the base of the calculation leads to the following floxuridine loads for each particle solution: The particles took up 14.7% of the initially provided floxuridine when loading pure floxuridine, 12.4% when loading with a 1:3 mixture of irinotecan and floxuridine and 6.9% when loading with a 1:1 mixture of irinotecan and floxuridine. This leads to a maximum floxuridine loading capacity of 3.61 wt% of the particles.

Table 1: Floxuridine loading for the different investigated particle solutions.

	Pure floxuridine	I:F 1:3	I:F 1:1
Loading (µg/mg Lip-MIL-88A)<br>(% of provided floxuridine that was taken up)	36.1 (14.7%)	22.8 (12.4%)	8.50 (6.9%)

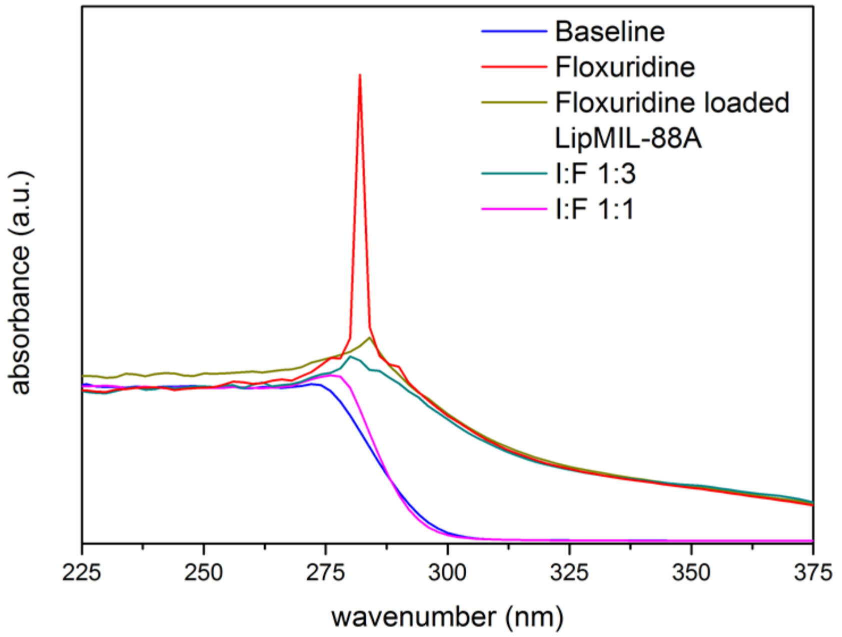

Figure S4: UV/Vis measurement of LipMIL-88A loaded with floxuridine and mixtures of floxuridine and irinotecan.

UV/Vis of irinotecan

In addition to the irinotecan measurements provided in the main text, we also measured the supernatant of Lip-MIL-88A loaded with irinotecan one week after preparation to check for potential leakage. There was no observable leakage of irinotecan from Lip-MIL-88A after one week (Figure S5).

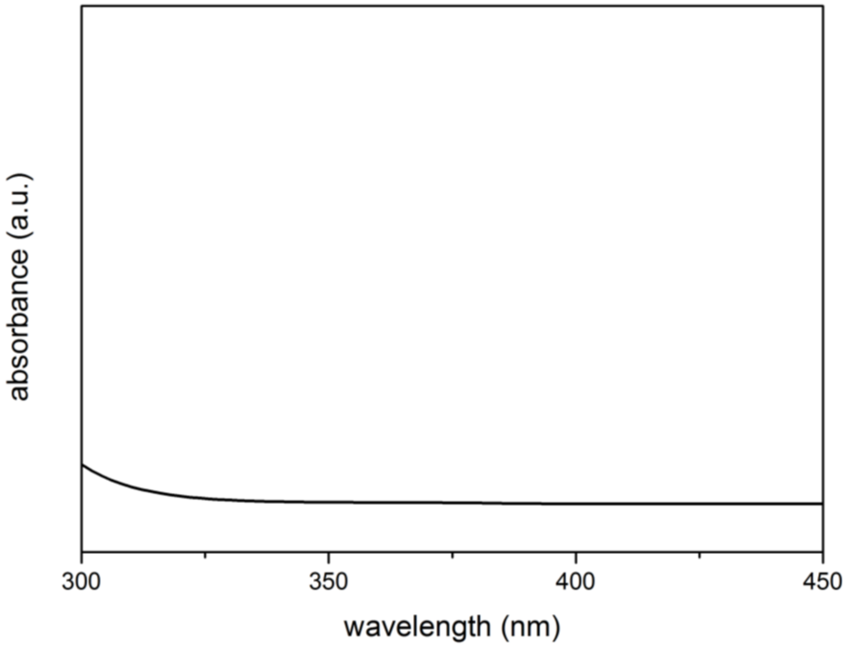

Figure S5: UV/Vis of 1 mg/mL Lip-MIL-88A loaded with irinotecan in PBS one week after preparation showing no leakage into the supernatant.

DLS

The DLS measurements show that both the pure and the liposome coated particles have an average size of about 100 nm, with the coated ones being slightly larger. The PDI is 0.153 and 0.143 respectively. The Zeta potential of the particles is −21.0 mV without liposome coating and 26.0 mV with liposome coating at pH 7.4.

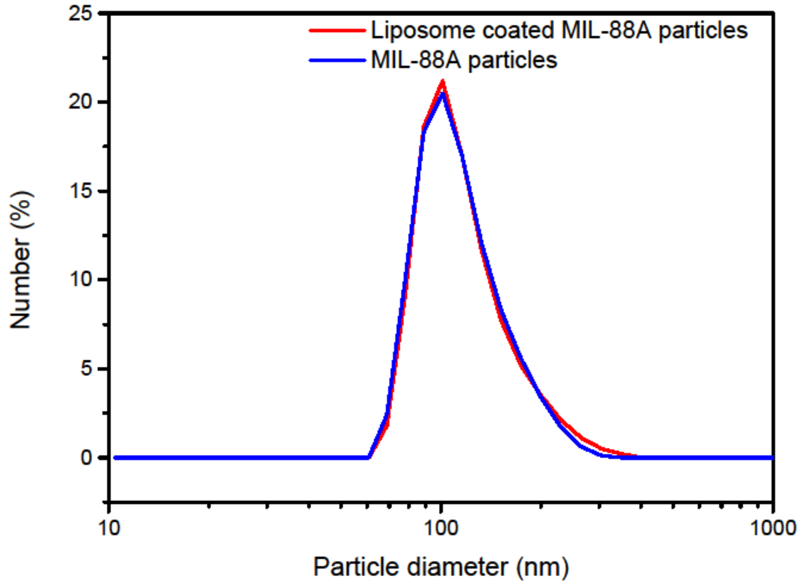

Figure S6: DLS measurements of MIL-88A nanoparticles (**blue**) and liposome coated MIL-88A nanoparticles (**red**).

Visualizing the Liposome Coating

To investigate if the MIL-88A NPs were successfully coated with liposomes, we performed fluorescence colocalization experiments. The MOF nanoparticles were labeled with Fluo-3 (0.2 µL Fluo-3 solution per 100 µL particle solution, in PBS; Figure S7, a), which stains the iron contained in the particles, while the liposomes were labeled with DiD (0.2 µL DiD solution per 100 µL particle solution, in PBS; Figure S7, b). A successful coating of the particles should result in a colocalization of both dyes,

while a failure in coating the particles should result in an independent distribution. The merged image (Figure S7, c) of both channels shows that we could observe the colocalization of both dyes suggesting a successful coating. The slight movement of the particles due to Brownian motion is preventing a complete overlap of both images in the merged channel as it takes time to switch between the different excitation wavelengths needed for the two different dyes. Control experiments with both dyes and only liposomes or MIL-88A particles showed no colocalization of the dyes, corroborating that the colocalization is due to the successful coating of the particles.

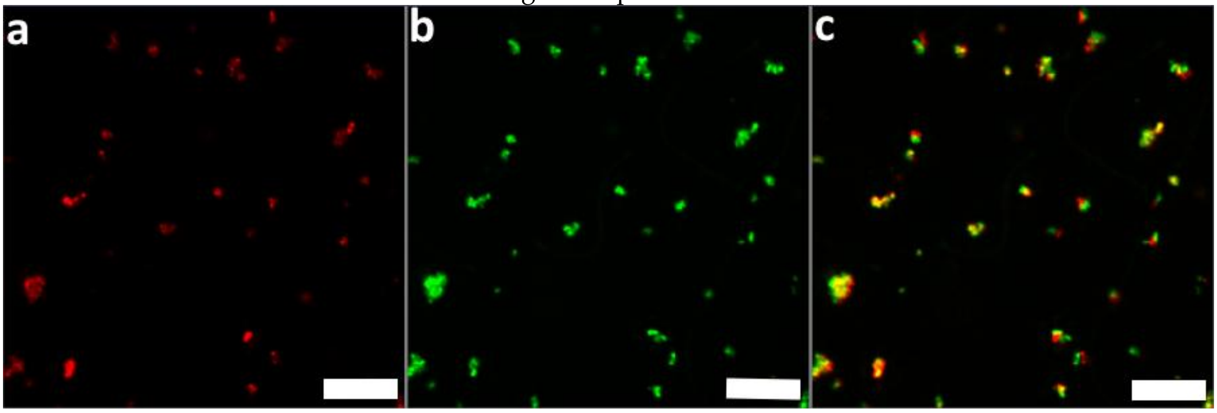

Figure S7: (**a**) MIL-88A marked with Fluo-3. (**b**) Liposomes marked with DiD. (**c**) Merged channels of (**a**) and (**b**). The scale bars in all images represents 50 µm.

Quantification through High-Content Experiments

The release behavior of the Lip-MIL-88A was further quantified by incubating different amounts of calcein loaded Lip-MIL-88A (0, 10, …, 140, 150 mg/L) in HeLa cells and counting the number of cells that show a visible release. For this 5000 HeLa cells were seeded in each well of a 96 well plated together with 100 µL DMEM (10%FBS, 1% PenStrep). After one day of incubation (37 °C, 5% CO_2) the particles were added. The images were taken after three and four days of incubation. Four images per well were taken and each concentration was measured in triplicate. The images have been evaluated with the MetaXpress software, using the built in functions and a custom module. First the total number of cells in the brightfield images were counted, then the cells showing full fluorescence in the GFP channel were also counted. To investigate the accuracy of the automated counts some of the images were evaluated by hand for a comparison.

By comparing it with the total number of cells the release efficiency of the drug carrier can be estimated. The number of cells that exhibit a successful release of the cargo after 3 to 4 days of incubation steadily grows with increasing concentration until a plateau is reached at a particle concentration of 110 µg/mL. The maximum of cells that exhibit a successful release of the cargo is between 50 and 60% regardless of the incubation time (Figure S8). The difference in incubation time makes no large difference in the cells that show visible release. In the microscope images we could observe that there were cells that also took up Lip-MIL88A, but did not show any signs of release even after four days of incubation (Figure S9). One possible reason for this behavior could be that these cells are currently at different stages in the cell cycle than the others.

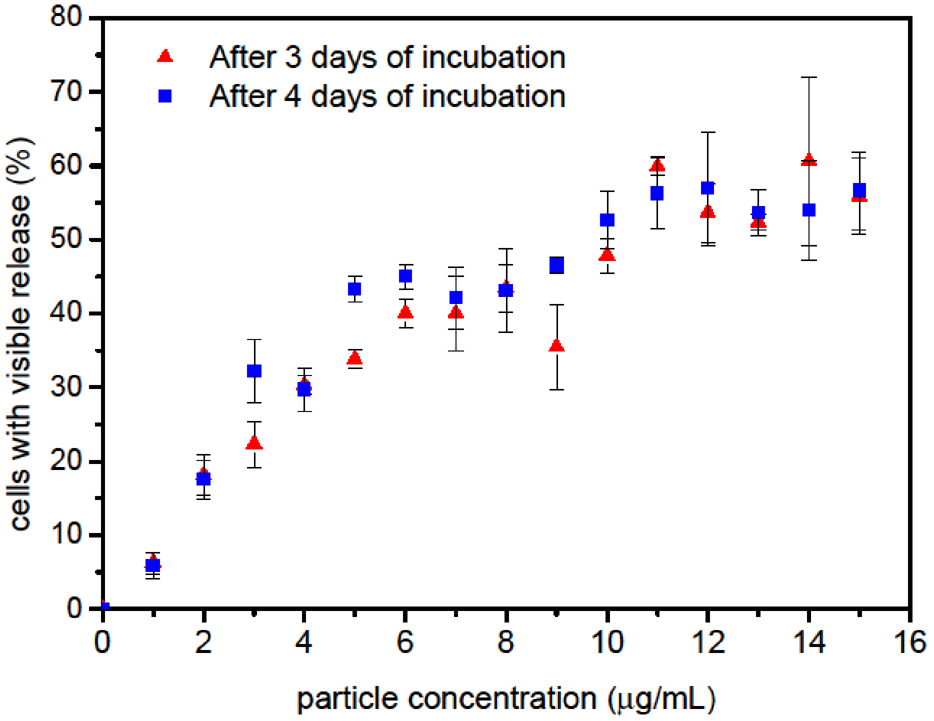

Figure S8: Quantification of the release of Lip-MIL-88A. Presented are the cells with visible signs of release for each concentration after 3 (**blue**) and 4 (**red**) days of incubation. The error bars represent SD.

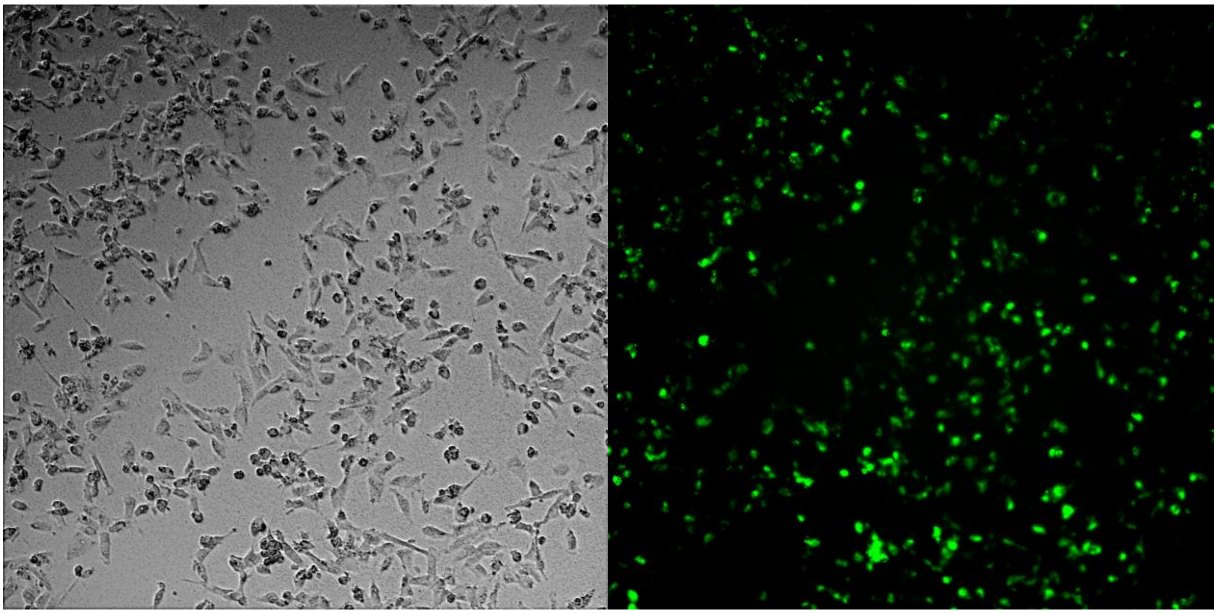

Figure S9: Sample images of the High Content Release Quantification of HeLa cells incubated for 3 days with particle concentration of 2 µg/mL. (Brightfield image on the **left** and the corresponding GFP channel image on the **right**)

HDAC1 Assay to determine the SBHA loading

SBHA is difficult to detect with standard analytical techniques. For this reason another quantification method had to be used. SBHA is an inhibitor of HDAC1 and we used its inhibition as a quantifier. The assay kit used was the HDAC1 Inhibitor Screening Assay Kit from Cayman Chemical. For the quantification different dilutions of SBHA were prepared and tested on their inhibition of HDAC1 and then compared to the inhibition of dissolved SBHA loaded particles to determine how much SBHA was loaded into the particles (Figure S10).

We calculated the theoretical dilution at 50% inhibition for SBHA (1:43) and the SBHA loaded MIL-88A NPs (1:6.1) from the experiment. From these dilutions and the concentrations of the stock solutions we could calculate how much SBHA (4.75 µg) and SBHA loaded particles (163 µg) were needed to inhibit 50% of the available HDAC1. We can then calculate the SBHA loading capacity of Lip-MIL-88A: 14.2 % of the initially available 1 mM SBHA were loaded into the particles or 2.90 wt% in relation to the weight of the MOF NPs.

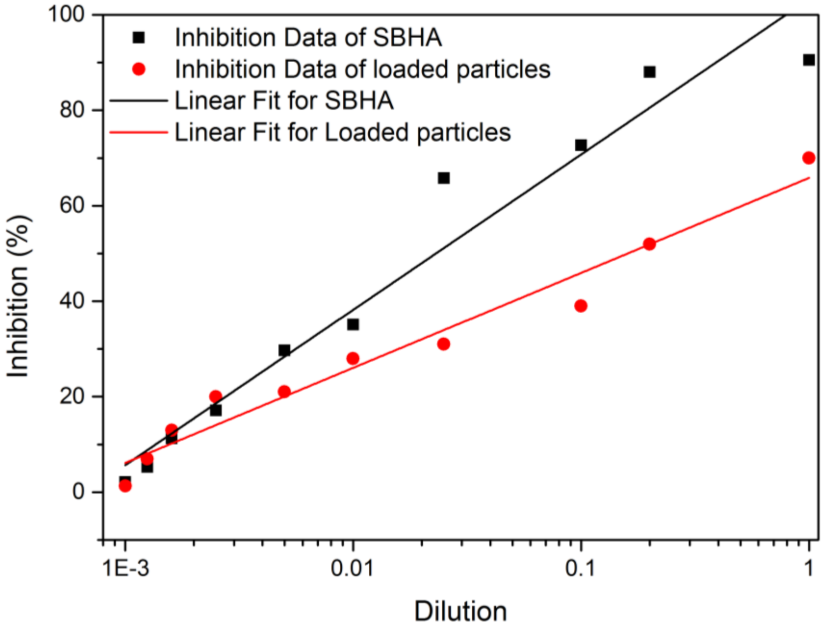

Figure S10: Graphical representation of the results of the HDAC1 Inhibition Assay.

MTT-Assays

To confirm that the Lip-MIL-88A shows no leakage and a good release of its cargo MTT tests were performed. For this purpose 5000 HEK 293T or HeLa cells were seeded into each well of a 96-Well Plate with 0.1 mL of DMEM. After one day of incubation the Lip-MIL-88A were added. The MTT test was performed after three days of incubation with the particles.

Several different therapeutic drugs and different concentrations of drug loaded Lip-MIL-88A were investigated. For the therapeutic drugs the doses were: 0, 0.2, 0.4, 0.8, 2, 6, 10, 14 µL/mL of a 1 mM stock solution. For Lip-MIL-88A the concentrations were: 0, 20, 40, 80, 20, 60, 100, 140 µg/mL. Each concentration was tested on three different days and in triplicate on each day. As a comparison to the drug loaded Lip-MIL-88A particles the cells were directly exposed to different concentrations of the pure drugs (Figure S11). As can be seen the drugs show low efficacy for drug concentrations below 2 µL/mL with cell viabilities of more than 60%, but lead to a strong decrease in cell viability for higher doses, resulting in cell viabilities below 10% for each drug with the highest tested concentration.

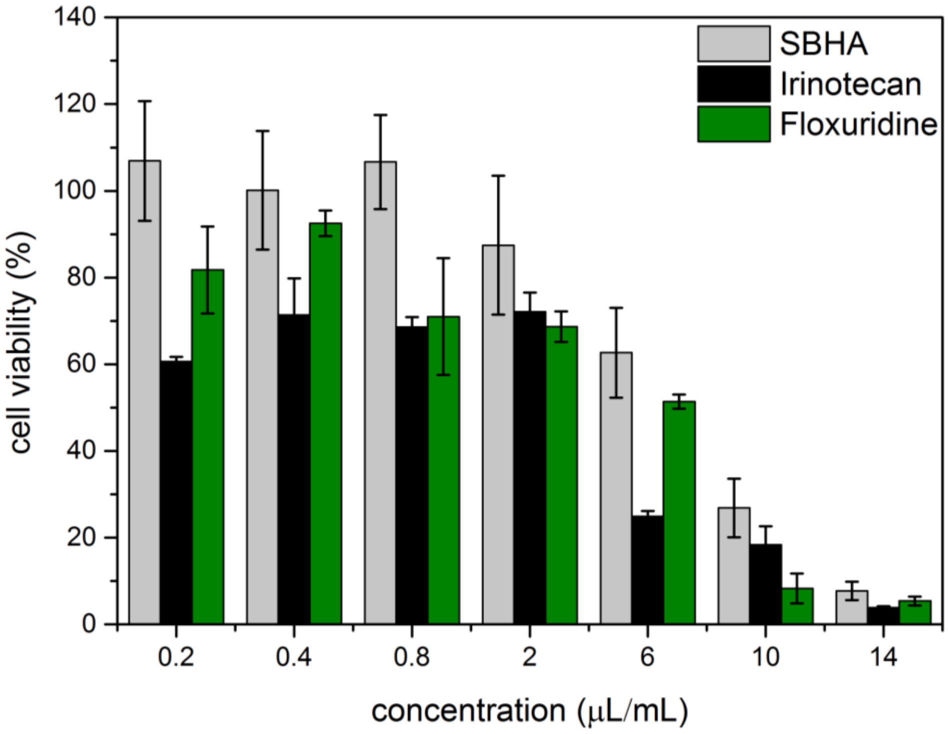

Figure S11: MTT-Assays of free drugs incubated for three days with HeLa cells.

MTT-Assays of the uncoated and the liposome coated MIL-88A NPs show that they are non-toxic with cell viabilities above 70% (Figure S12).

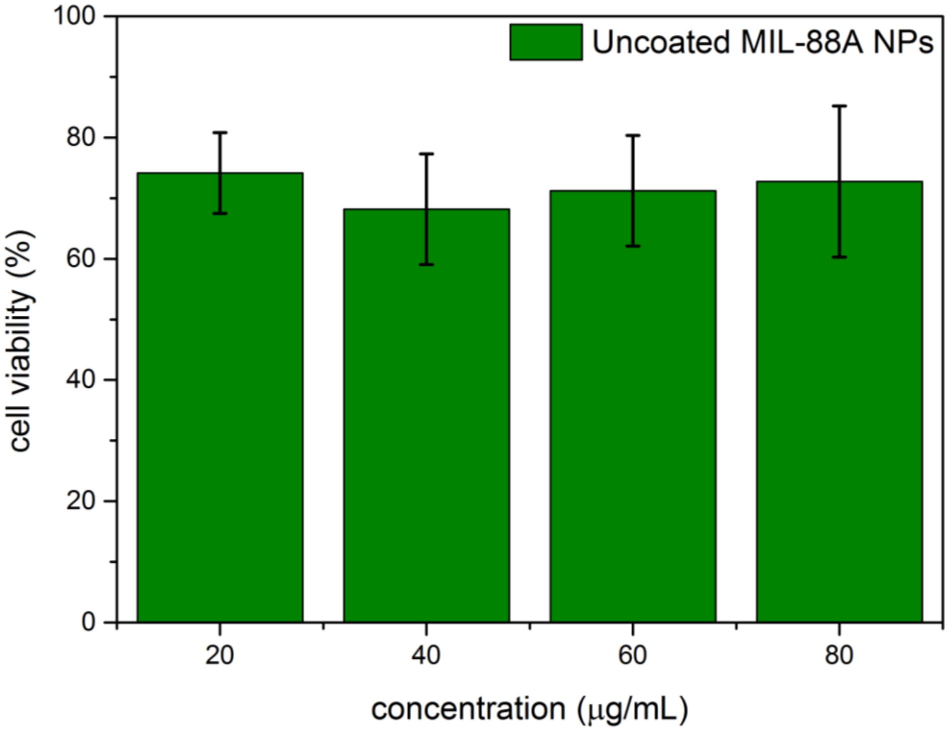

Figure S12: MTT-Assay of Uncoated MIL-88A NPs.

Figure S13 shows the MTT-Assay of Lip-MIL-88A without any cargo inside. The particles were incubated for three days in HeLa cells and show no toxicity with cell viabilities above 90%.

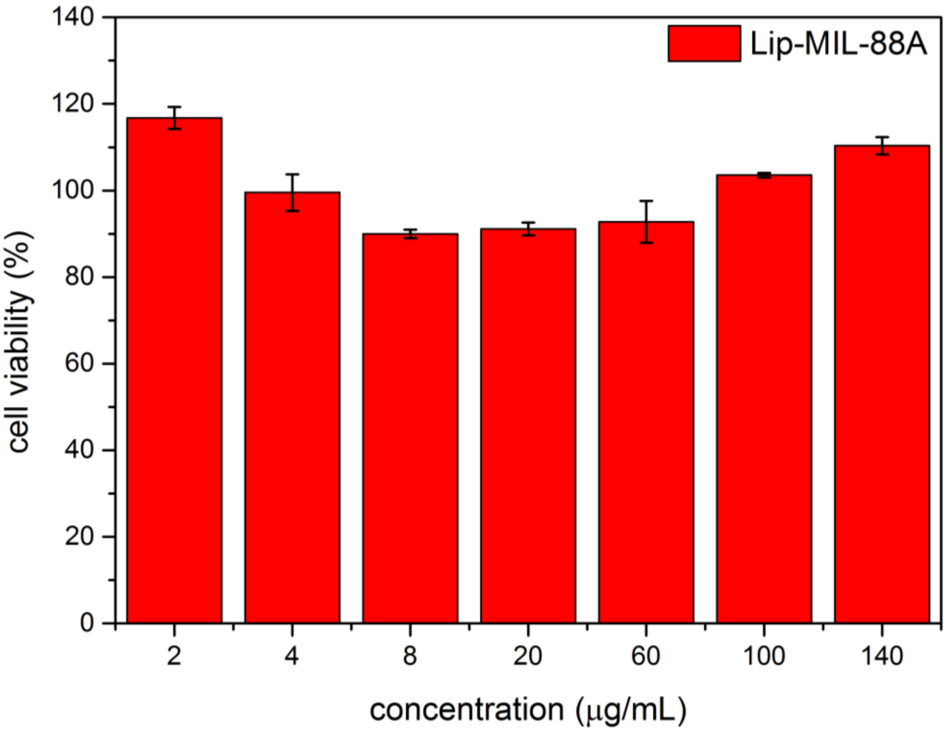

Figure S13: MTT Assay of liposome coated MIL-88A NPs without cargo.

References

1. Andrew Lin, K.-Y.; Chang, H.-A.; Hsu, C.-J. Iron-based metal organic framework, MIL-88A, as a heterogeneous persulfate catalyst for decolorization of Rhodamine B in water. *RSC Advances* **2015**, *5*, 32520-32530.
2. McKinlay, A.C.; Eubank, J.F.; Wuttke, S.; Xiao, B.; Wheatley, P.S.; Bazin, P.; Lavalley, J.C.; Daturi, M.; Vimont, A.; De Weireld, G., *et al.* Nitric oxide adsorption and delivery in flexible MIL-88(Fe) metal-organic frameworks. *Chem. Mater.* **2013**, *25*, 1592-1599.
3. Illes, B., Hirschle, P., Barnert, S., Wuttke, S, Engelke, H, Exosome-Coated Metal-Organic Framework Nanoparticles: An Efficient Drug Delivery Platform, *Chem. Mater.* **2017**, 29, 8042–8046, Doi: 10.1021/acs.chemmater.7b02358.
